# Discrepancies between two long-term dietary datasets in the United Kingdom (UK)

**DOI:** 10.12688/wellcomeopenres.17245.1

**Published:** 2021-12-16

**Authors:** Kerry G. Smith, Pauline Scheelbeek, Andrew Balmford, Emma E. Garnett

**Affiliations:** 1 Department of Zoology, University of Cambridge, Cambridge, CB2 3EJ, UK; 2 Centre on Climate Change & Planetary Health, London School of Hygiene & Tropical Medicine, London, WC1E 7HT, UK; 3 Cambridge Institute for Sustainability Leadership, University of Cambridge, Cambridge, CB2 1QA, UK

**Keywords:** Dietary change, food balance sheets, household budget survey, UK, longitudinal

## Abstract

**Background: **Studying dietary trends can help monitor progress towards healthier and more sustainable diets but longitudinal data are often confounded by lack of standardized methods. Two main data sources are           used for longitudinal analysis of diets: food balance sheets on food supply (FBS) and household budget surveys on food purchased (HBS).

**Methods: **We used UK longitudinal dietary data on food supply, provided by the Food and Agriculture Organisation (FAO) (FAO-FBS, 1961-2018), and food purchases, provided by Defra (Defra-HBS, 1942-2018). We assessed how trends in dietary change per capita compared between FAO-FBS and Defra-HBS for calories, meat and fish, nuts and pulses, and dairy, and how disparities have changed over time.

**Results: **FAO-FBS estimates were significantly higher (p<0.001) than Defra-HBS for calorie intake and all food types, except nuts and pulses which were significantly lower (p<0.001). These differences are partly due to inclusion of retail waste in FAO-FBS data and under-reporting in Defra- HBS data. The disparities between the two datasets increased over time for calories, meat and dairy; did not change for fish; and decreased for nuts and pulses . Between 1961 and 2018, both FAO-FBS and Defra-FBS showed an increase in meat intake (+11.5% and +1.4%, respectively) and a decrease in fish (-3.3% and -3.2%, respectively) and dairy intake (-11.2% and -22.4%). Temporal trends did not agree between the two datasets for calories, and nuts and pulses.

**Conclusions: **Our finding raises questions over the robustness of both data sources for monitoring UK dietary change, especially when used for evidence-based decision making around health, climate change and sustainability.

## Introduction

Monitoring dietary trends is important for measuring progress towards healthier and more sustainable diets. While several longitudinal monitoring databases exist, a number of challenges limit their usefulness for analysis including scarcity of standardized methods ( De Keyzer *et al*., 2015; Perignon *et al*., 2017), lack of waste monitoring ( Whybrow *et al*., 2017) and variation in the stages of food production being measured ( Bandy *et al*., 2019; Serra-Majem *et al*., 2003). Both food balance sheets (FBS) (published by Food and Agriculture Organisation (FAO), hereafter referred to as FAO-FBS) and household budget surveys (HBS) (published by Defra in the United Kingdom (UK), hereafter referred to as Defra-HBS) have been used to approximate trends in consumption over time (for example Grünberger, 2014 and Peng *et al*., 2015).

FBS measure food consumption from a food supply perspective and considers domestic production, imports and exports. The advantage of FBS over HBS is that they are produced in a standardised format, which facilitates comparison between countries, and the combination of food data with associated statistics on trade and agricultural practices ( Balanza *et al*., 2007; Del Gobbo *et al*., 2015). However, despite adjustments, FAO (2017) report that these FBS can be incomplete or unreliable due to gaps and inaccuracies in underlying data, and complexities in transforming data from a diverse range of sources into a standardised format. HBS measure food consumption from a food purchase perspective ( FAO, 2001). Alongside the HBS published by Defra since 1942, Public Health England and the UK Food Standards Agency have conducted the National Diet and Nutrition Survey since 2008-09 ( Public Health England, 2020). Here we use the HBS surveys produced by Defra due to the long time period of their operation (81 years compared to the 13 years of the National Diet and Nutrition Survey), permitting the analysis of long-term temporal trends. The strength of HBS is that data are gathered alongside demographic information, which enables the study of consumption characteristics. FBS and HBS measure food consumption at a different stage of the food supply chain: FBS record the quantity of food that reaches shops and other food outlets, while HBS record the quantity of food that is bought. Because of this, FBS include retail waste in quantity of food supply, whereas HBS do not. Both FBS and HBS estimates include waste at the household level, so overestimate the quantity of food actually eaten. In the UK, 70% of post-farm-gate waste occurs in households ( WRAP, 2021). When conducting HBS, reported food purchases are often less than the actual quantity of food bought due to participants’ under-reporting ( Mendez *et al*., 2004; Office for National Statistics, 2016).

To our knowledge, the discrepancies between FBS and HBS for monitoring dietary change have never been quantified for the UK. However, as both data sources play a pivotal role in providing evidence for decision-making, it is important that these discrepancies are mapped, and their implications for evidence generation are known. Findings from studies outside the UK indicate that using only one of these methodologies to assess dietary trends can be highly problematic ( Del Gobbo *et al*., 2015; Serra-Majem *et al*., 2003), and investigating reasons behind data discrepancies helps to understand their limitations ( Benthem de Grave *et al*., 2020). In this study, we compared time-series FBS data produced by FAO (FAOSTAT, 1961–2018, FAO-FBS) on UK food supply to HBS data produced by Defra (National Food Survey, 1942–2000 and Family Food Module, 2000–2018, Defra-HBS) on UK food purchases to determine to what extent the data sources agree, both on overall calorie intake and specific food groups. We assessed how agreement between FAO-FBS and Defra-HBS has changed between 1961 and 2018 (the most up to date data currently available) and the relevance of differences between FAO-FBS and Defra-HBS for evidence-based decision making.

## Methods

### Secondary datasets

**Figure 1.**
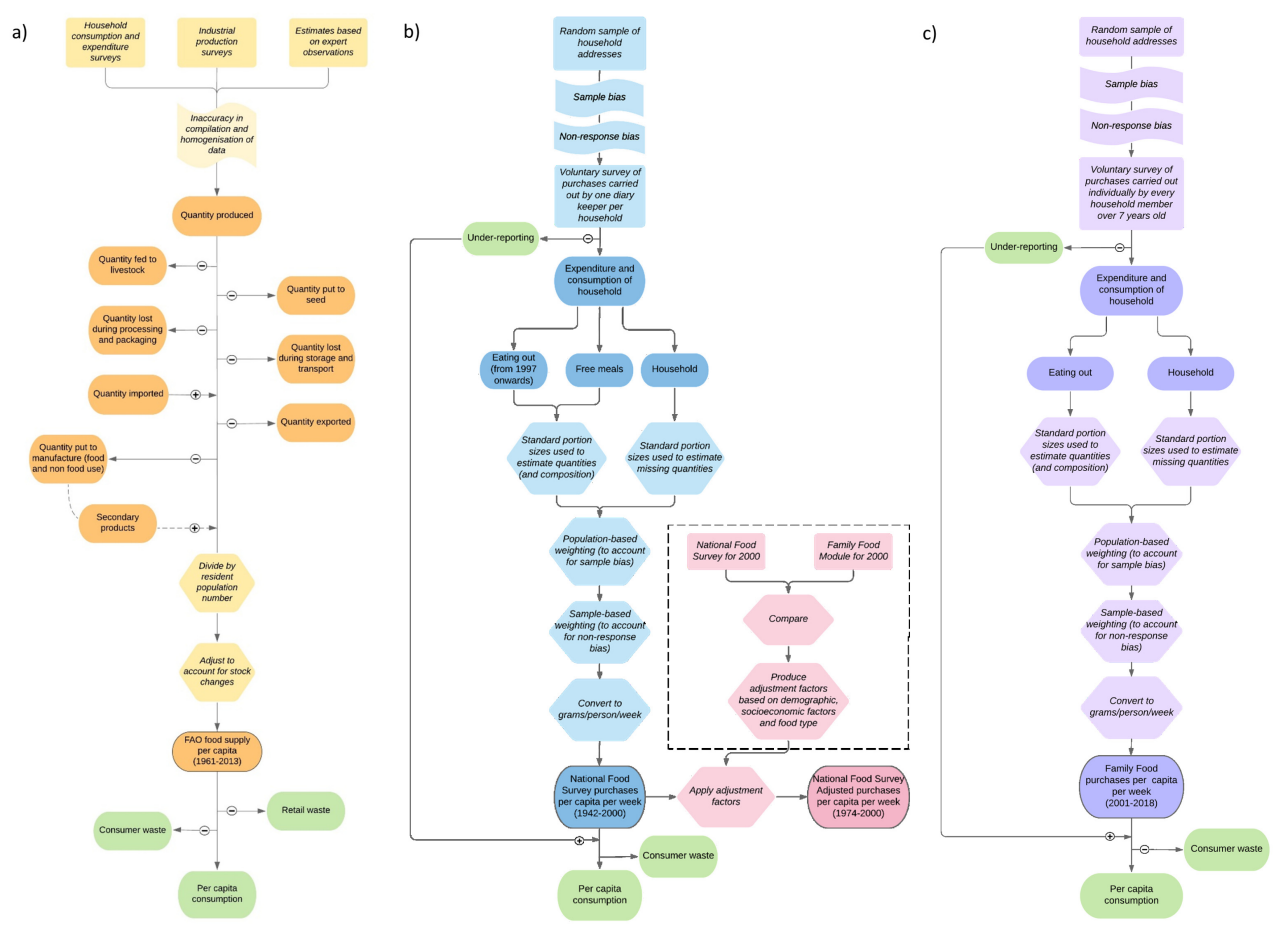
Methodology of **a**) FAOSTAT (Food and Agriculture Organisation food balance sheets (FAO-FBS)), **b**) National Food Survey and adjusted National Food Survey (Defra-household budget surveys (Defra-HBS)) and **c**) Family Food Module (Defra-household budget surveys). Orange ( **a**), blue ( **b**), pink ( **b**) and purple ( **c**) boxes show data values and processes involved in data collection and processing. Green boxes show values not measured. Dark shaded oblongs indicate data values, light shaded oblongs show processes involved in data gathering, hexagons indicate the steps involved in handling data and flags show biases in data gathering.

FAO-FBS consist of compiled, cleaned and standardised data from national statistics on food supply – the quantity of food available to buy per person. Data for food balance sheets are gathered from a number of sources, including industrial production surveys, estimates based on expert observations and household and expenditure surveys ( FAO, 2001). FAO adjusts the basic data to account for biases and inaccuracies in data reporting and estimate missing data. We made no further adjustments to account for data inaccuracies and biases, and used the data as provided by the FAO. FAO updated their methodology in 2014. The primary change was the shift from using 2015 United Nations Development Programme (UNDP) population data (used before 2014) to using updated 2019 UNDP population data ( FAO, 2020). As some of the revised population numbers are higher than those used previously this can affect per capita values. Despite this, the methodology of FAO-FBS before and after 2014 is broadly consistent so is treated as a single, continuous dataset. Figure 1a summarises the overall method ( FAO, 2001; FAO, 2017; Ritchie & Roser, 2020). FAO-FBS allows comparison between countries as data are produced in a standardised format on nearly all food products.

We compared these FAO-FBS with Defra-HBS, which consists of three household datasets: The National Food Survey, the adjusted National Food Survey and the Family Food Module. All are HBS that record purchases over time of the quantity of food and drink bought by a household. The National Food Survey and Family Food Module use a stratified random sample (with clustering) of UK households ( Defra, 2012). In 2011, 5692 households were sampled. Surveys are spread out throughout the year to ensure seasonal effects are accounted for. Both include a voluntary survey in which purchases of food and drink (after 1992) are recorded over a two-week period. Defra make adjustments to account for sampling bias and non-response bias. We made no further adjustments to account for data inaccuracies and biases, and used the data as provided by Defra. To account for differences between the National Food Survey and the Family Food Module methodology, adjusted quantities of the National Food Survey were produced by Defra for 1974 to 2000 ( Defra, 2011). Defra-HBS data collection methodologies are summarised in Figure 1b and c ( Defra, 2011; Defra, 2012). The difference between Defra-HBS methodologies was deemed small enough to treat all three datasets as continuous. All data on supply and purchases are expressed in grams/capita/day other than calorie intake which is expressed in kcal/capita/day.

As this study did not involve human subjects and used open-source secondary data which did not include any personal information, an independent ethical review was not required.

The datasets used in this study can be found in the *Data availability* section ( Smith *et al*., 2021).

### Data analysis

Data analysis was carried out in Excel Version 2108 and R version 3.6.1 ( R Core Team, 2020). Data were converted from kg/capita/year to g/capita/day then aggregated according to food types ( Table 1). We calculated mean daily per capita supply and production of meat and fish, dairy, nuts and pulses, and calories for each year covered by each data source ( Table 1). No data were removed during the analysis. Defra-HBS cheese was converted to milk equivalent by multiplying mass (g) by 10 (the extraction rate from milk to cheese is 10% in the United Kingdom [ FAO, 2000]). Defra-HBS milk and products were converted from millilitres to grams (using the density of cow’s milk reported by Cziszter *et al*., 2012 [1.03g/cm ^3^]). The food types analysed were chosen as they are produced in categories which are comparable between the two data sources, and provide an indication of changes in total consumption (calorie intake) and protein intake (meat, fish, dairy, nuts and pulses represent the majority of total protein intake in the UK [ British Nutrition Foundation, 2018]). Differences in aggregation of food categories between FAO-FBS and Defra-HBS makes comparison between some food types challenging. For example, Defra-HBS report purchases of bread, whereas FAO-FBS report supply of wheat and products. As many food groups are produced in aggregated categories, converting to nutritional intake can be difficult ( Serra-Majem *et al*., 2003). Whilst data on supply and purchases of selected macronutrients are provided by FAO-FBS and Defra-HBS, here we have compared data on food groups between the two data sources, as data on food supply and purchases have been used to assess how diets ( Serra-Majem *et al*., 2003; Thar *et al*., 2020), and their associated health ( Aiello *et al*., 2019) and environmental impacts ( Lucas *et al*., 2021) have changed.

**Table 1.** Methods for estimating household-level consumption of different food types (rows) from Defra (National Food Survey, National Food Survey Adjusted and Family Food Module) and FAO (Food and Agriculture Organisation) sources (columns). Defra data on nuts and pulses purchases are only available from 1974 onwards. *Margarine was excluded from dairy purchases of National Food Survey (Defra). Family food module: hh= household; eo= eating out.

Food type	National Food Survey (Defra 1942- 1974) “Household consumption of selected foods from 1942 to 2000”	National Food Survey Adjusted (Defra 1974-2000) “UK-household purchases”	Family Food Module (Defra 2000-2018) “UK-household purchases” and “UK- eating out purchases”	FAO (1961-2018) (food supply quantity)
Meat	Total meat and meat products + Total fish and fish products	Carcase meat + Non- carcase meat and meat products + Fish	Carcase meat ^hh^ + Non-carcase meat and meat products ^hh^ + Meat and meat products ^eo^ + Fish ^hh^ + Fish and fish products ^eo^	Meat (total) + Fish, seafood (total)
Dairy	Total milk and cream + Total cheese + Butter	Milk products and milk products excluding cheese + Cheese	Milk products and milk products excluding cheese ^hh^ + Cheese ^hh^ + Milk-based drinks ^eo^ + Cheese ^eo^ + Yoghurt and fromage frais ^eo^ + Ice cream ^eo^	Milk- Excluding Butter + Butter, Ghee + Cream
**Nuts and** **Pulses**	No data	Nuts, seeds and peanut butter + Dried pulses other than air-dryed + Other canned beans and pulses	Nuts, seeds and peanut butter ^hh^ + Dried pulses other than air-dryed ^hh^ + Other canned beans and pulses ^hh^ + Beans and pulses ^eo^ + Nuts and seeds ^eo^	Beans + Groundnuts (Shelled Eq) + Nuts and products + Pulses, other and products + Soyabeans (available at http://www.fao.org/faostat/ en/#data/CC)
**Calories**	Energy (kcal) Data from “Household nutrient data from 1940 to 2000 – 1940- 2000”	Energy (kcal) Data from “ UK - household and eating out nutrient intakes (Household_ intake)”	Energy (kcal) Data from “UK - household and eating out nutrient intakes (Total_intake)”	Grand total (food supply, kcal/capita/day)
Bovine	Beef and veal	Beef and veal + Ox liver + Corned beef, canned or sliced	Beef and vealhh + Ox liver ^hh^ + Corned beef, canned or sliced ^hh^ + Steak - without sauce (e.g. braised, sirloin) ^eo^	Bovine Meat
Mutton	Mutton and lamb	Mutton and lamb + lamb liver	Mutton and lamb ^hh^ + lambs liver ^hh^ + Lamb chops with sauce or gravy ^eo^	Mutton & Goat Meat
Pork	Pork, bacon and ham	Pork + Sausages, uncooked – pork + Bacon and ham, cooked + Bacon and ham, uncooked + Pigs liver	Pork ^hh^ + Sausages, uncooked – pork ^hh^ + Bacon and ham, cooked ^hh^ + Bacon and ham, uncooked ^hh^ + Pigs liver ^hh^ + Bacon ^eo^ + Pork chops with sauce or gravy ^eo^ + Gammon or ham ^eo^	Pigmeat
Poultry	Poultry	Cooked poultry not purchased in cans + Chicken, uncooked - whole chicken or chicken pieces + Other poultry, uncooked (including frozen)	Cooked poultry not purchased in cans ^hh^ + Chicken, uncooked - whole chicken or chicken pieces ^hh^ + Other poultry, uncooked (including frozen) ^hh^ + Poultry ^eo^ + Chicken burger ^eo^	Poultry Meat
Fish	Total fish and fish products	Fish	Fish ^hh^ + Fish and fish products ^eo^	Fish, seafood (total)

We fitted a linear model for FAO-FBS food supply against time (1961–2018) for all food types and calorie provision to analyse long term trends in the UK diet. We repeated this for Defra-HBS food purchases against time (1942–2018). Ruminant supply and purchases were calculated by summing supply and purchases of beef and mutton (lamb). Gradient of slope was compared between FAO-FBS and Defra-HBS (mean change, g/capita/day per year).

For each year (1961–2018) we calculated the difference between FAO-FBS food supply and Defra-HBS food purchases (as a percentage of Defra-HBS purchases) for each food type in turn. We explored whether this difference changed over time using correlation tests: Pearson’s product-moment for normally distributed data, Spearman’s rank for non-normally distributed data, after assessing for normality using Shapiro-Wilk. Finally, we estimated the mean difference between FAO-FBS food supply and Defra-HBS food purchases across all years for each food category.

## Results

### Differences between FAO-FBS and Defra-HBS in per capita quantities of food

**Figure 2.**
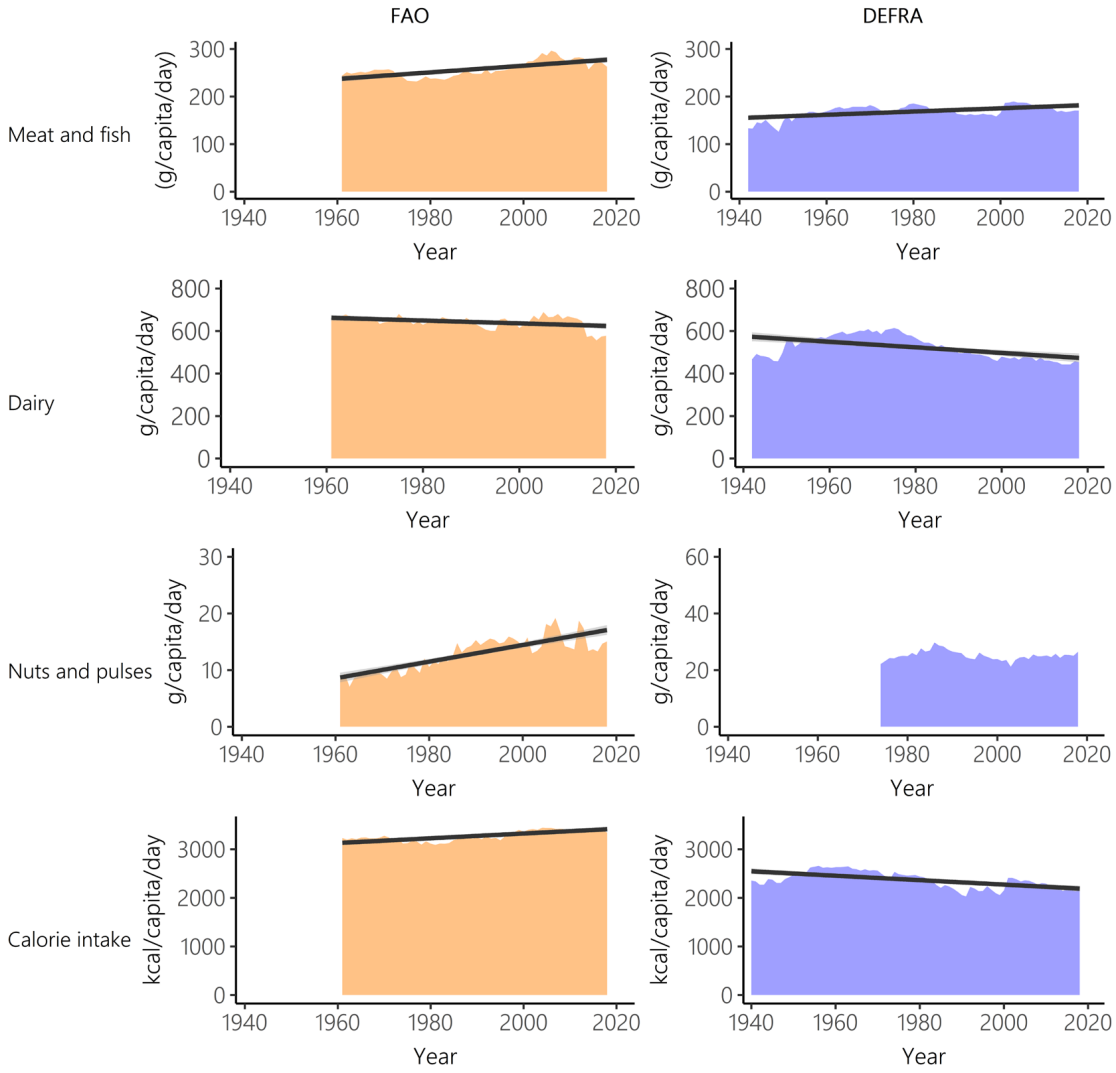
Meat and fish, dairy, nuts and pulses, and calorie intake over time for Food and Agriculture Organisation food balance sheets (FAO-FBS) food supply (orange) and Defra-household budget surveys (Defra-HBS) food purchases (blue). Linear model of supply/purchases over time (black) and 95% confidence intervals (grey). In all cases, evaluation of assumptions through use of diagnostic plots indicated no violations.

Comparison of long-term data on UK food supply from FAO-FBS (1961-2018) and food purchases from Defra-HBS (1942–2018) show many inconsistencies. Averaged across all years between 1961 and 2018 (1974–2018 for nuts and pulses), FAO-FBS food supply data reported significantly higher per capita outputs than Defra-HBS food purchases for meat and fish (V=0, n=58, p<0.001), dairy (V=1711, n=58, p<0.001) and calorie consumption (V=1711, n=58, p<0.001). On average, FAO-FBS meat and fish per capita outputs were 48% higher than Defra-HBS, FAO-FBS dairy per capita outputs were 24% higher than Defra-HBS and FAO-FBS calorie provision was 41% higher than Defra-HBS ( Figure 2). This contrasts to nuts and pulses where FAO-FBS food supply was significantly lower (44%) than Defra-HBS food purchases (V=0, n=45, p<0.001).

### Temporal trends: food type intake

FAO-FBS and Defra-HBS datasets show different long-term trends in UK consumption ( Table 3). Between 1961 and 2018, both data sources do show increasing intake of meat (FAO-FBS, +11.5%; Defra-HBS, +1.4%) and decreasing intake of fish (FAO-FBS, -3.3%; Defra-HBS, -3.2%) and dairy (FAO-FBS, -11.2%; Defra-HBS, -22.4%) though the magnitude of change generally differs substantially. However, FAO-FBS shows an increase in calorie consumption (1961–2018, +3.5%) while Defra-HBS records a decrease (1961–2018, -17.3%). Per capita supply of nuts and pulses and calorie provision show a clear increasing trend when based on FAO-FBS, while the purchase data from Defra-HBS show a stable per capita purchase pattern ( Figure 2 and Table 2).

**Table 2.** Linear model outputs of FAO-FBS (Food and Agriculture Organisation food balance sheets) food supply (1961-2018) and Defra-HBS (household budget surveys) food purchases (1942-2018) against time for all food types and calories.

	FAO-FBS food supply (1961-2018)		Defra-HBS food purchases (1942-2018)	
**Food type**	p value	mean change (g/capita/day per year)	p value	mean change (g/capita/day per year)
Meat and fish	<0.001	0.70	<0.001	0.34
Dairy	<0.001	-0.67	<0.001	-1.31
Nuts and pulses	<0.001	0.12	>0.05	-0.02
Calories	<0.001	4.89 (kcal/capita/day per year)	<0.001	-4.56 (kcal/capita/day per year)
Ruminant	<0.001	-0.66	<0.001	-0.59
Pork	<0.001	-0.11	<0.001	<0.01
Poultry	<0.001	1.42	<0.001	0.59
Fish	>0.05	0.04	<0.001	<0.01

Disaggregating total meat and fish by individual meat type, gives further detail on the different trends in supply and purchases. According to FAO-FBS, total meat consumption increased by 11.5% between 1961 and 2018 and decreased by 5.2% between 2008 and 2018; Defra-HBS estimated a 1.4% increase and a 3% decrease respectively. FAO-FBS estimated a 6.1% decrease in fish consumption between 1961 and 2018, and a 12.5% decrease between 2008 and 2018; Defra-FBS estimated a 13.9% decrease for both time intervals ( Table 2 and Table 3).

**Figure 3.**
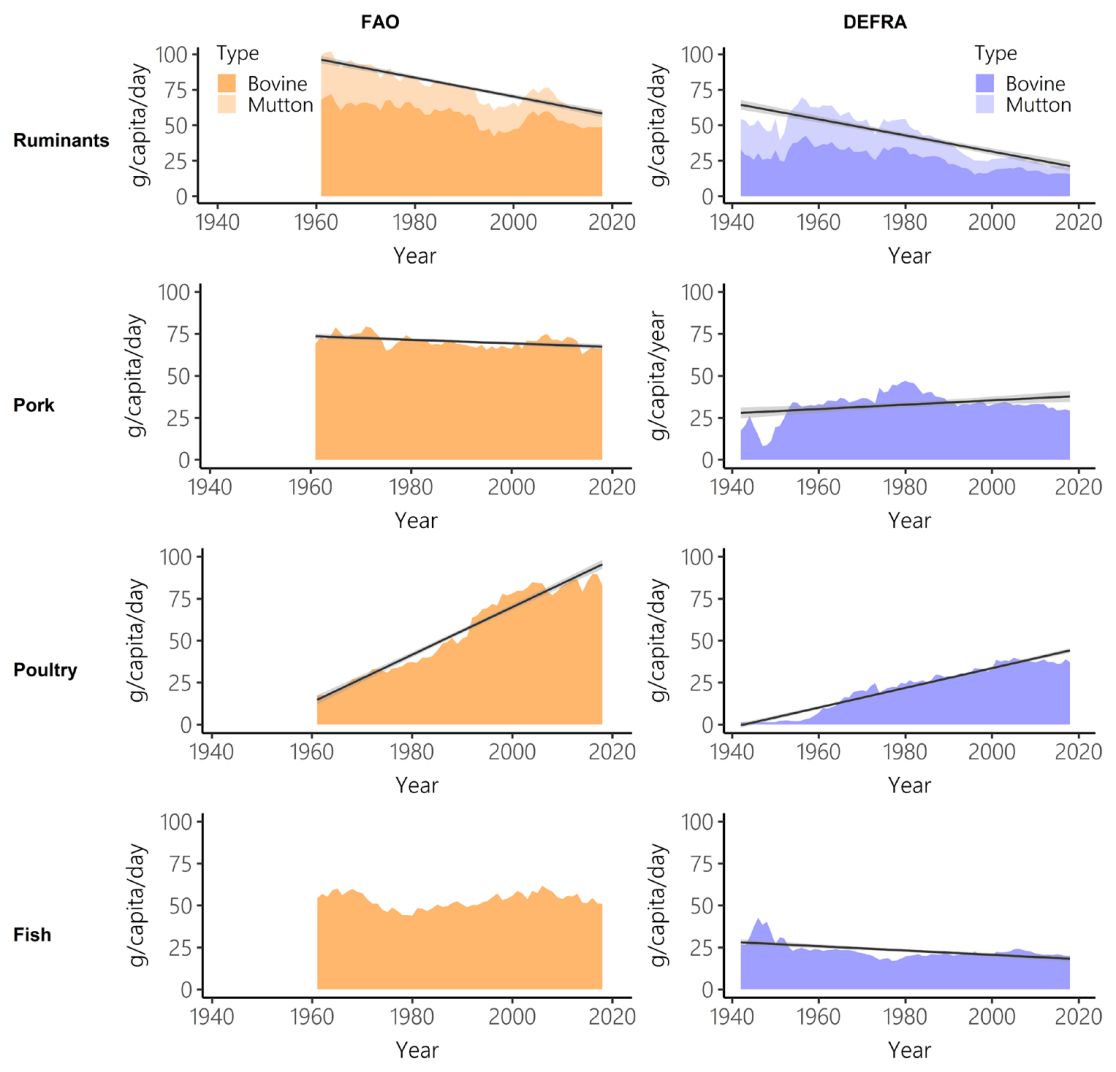
Ruminant, pork, poultry and fish intake over time for Food and Agriculture Organisation food balance sheets (FAO-FBS) food supply (orange) and Defra household budget surveys (Defra-HBS) food purchases (blue). Linear model of supply/purchases over time (black) and 95% confidence intervals (grey). In all cases, evaluation of assumptions through use of diagnostic plots indicated no violations.

FAO-FBS and Defra-HBS both show a sharp decrease in ruminant intake and a steep increase in poultry consumption ( Figure 3, Table 2 and Table 3). Pork and fish show more stable consumption over time. Between 1961 and 2018, Defra-HBS estimated steeper falls in ruminant and pork consumption than FAO-FBS (ruminant: FAO-FBS -39.7%, Defra-HBS -67.4%; pork: FAO-FBS -4.3%, Defra-HBS -11.2%), and a smaller increase in poultry consumption (FAO-FBS, +381.4%; Defra-HBS +227.8%, Table 3). However, these results for ruminants, pork and poultry should be interpreted cautiously, as the Defra-HBS categorisations did not allow the total intake of these meat types to be calculated (for example, takeaways and meat pies could not be assigned), unlike FAO-FBS data ( Table 1).

**Table 3.** Intake of different food types every 10 years for FAO-FBS (Food and Agriculture Organisation food balance sheets) and Defra-HBS (household budget surveys). All units are grams/capita/day, except for calories which is kcal/capita/day. Ruminant, pork and poultry do not sum to Total Meat because of a) other meat such as game and b) unidentified meat reported in Defra-HBS, e.g. meat pies.

Food type	Data Source	1961	1968	1978	1988	1998	2008	2018	Change between 1961 and 2018 in grams (%)	Change between 2008 and 2018 in grams (%)
**Total meat** **and fish**	**FAO-FBS**	244	256.7	236.2	252.7	262.2	281.6	262.6	18.6 (7.6%)	-19 (-6.7%)
	**Defra-HBS**	171.8	178.9	177.6	172.4	161.9	178.5	170.7	-1.1 (-0.6%)	-7.8 (-4.4%)
***Total fish***	***FAO-FBS***	*54.3*	*59.9*	*44.3*	*51.5*	*53.2*	*58.3*	*51.0*	*-3.3 (-6.1%)*	*-7.3 (-12.5%)*
	***Defra-HBS***	*23*	*23.0*	*17.4*	*20.8*	*21.1*	*23.0*	*19.8*	*-3.2 (-13.9%)*	*-3.2 (-13.9%)*
***Total meat***	***FAO-FBS***	*189.7*	*189.7*	*196.8*	*191.9*	*201.2*	*209*	*223.3*	*21.9 (11.5%)*	*-11.7 (-5.2%)*
	***Defra-HBS***	*148.8*	*148.8*	*155.9*	*160.2*	*151.6*	*140.8*	*155.5*	*2.1 (1.4%)*	*-4.6 (-3%)*
***Ruminant***	***FAO-FBS***	*99.9*	*93.1*	*85.2*	*78.0*	*62.8*	*73.1*	*60.2*	*-39.7 (-39.7%)*	*-12.9 (-17.6%)*
	***Defra-HBS***	*64.1*	*54.6*	*53.7*	*40.4*	*25.1*	*24.4*	*20.9*	*-43.2 (-67.4%)*	*-3.5 (-14.3%)*
***Pork***	***FAO-FBS***	*69.3*	*74.4*	*71.2*	*71.2*	*67.9*	*71.5*	*66.3*	*-3 (-4.3%)*	*-5.2 (-7.3%)*
	***Defra-HBS***	*32.9*	*34.9*	*44.2*	*37.3*	*34.4*	*32.5*	*29.2*	*-3.7 (-11.2%)*	*-3.3 (-10.2%)*
***Poultry***	***FAO-FBS***	*17.2*	*26.8*	*34.7*	*51.6*	*77.9*	*77.1*	*82.8*	*65.6 (381.4%)*	*5.7 (7.4%)*
	***Defra-HBS***	*9.9*	*19.4*	*22.4*	*29.6*	*33.2*	*38.2*	*37.4*	*27.5 (277.8%)*	*-0.8 (-2.1%)*
**Dairy**	**FAO-FBS**	651.3	652.8	631.5	639.1	642.2	680.3	578.1	-73.2 (-11.2%)	-102.2 (-15%)
	**Defra-HBS**	584.2	599.9	580.7	520.4	465.5	459.5	453.2	-131 (-22.4%)	-6.3 (-1.4%)
**Nuts and** **pulses**	**FAO-FBS**	8.5	9.8	9.5	13.9	15.6	16.8	15.1	6.6 (77.6%)	-1.7 (-10.1%)
	**Defra-HBS**	NA	NA	24.8	28.4	24.5	25.5	26.5	NA	1 (3.9%)
**Calories**	**FAO-FBS**	3231	3223	3095	3248	3352	3422	3344	113 (3.5%)	-78 (-2.3%)
	**Defra-HBS**	2630	2560	2465	2188	2101	2276	2175	-455 (-17.3%)	-101 (-4.4%)

### Temporal trends: differences between FAO-FBS and Defra-HBS

**Figure 4.**
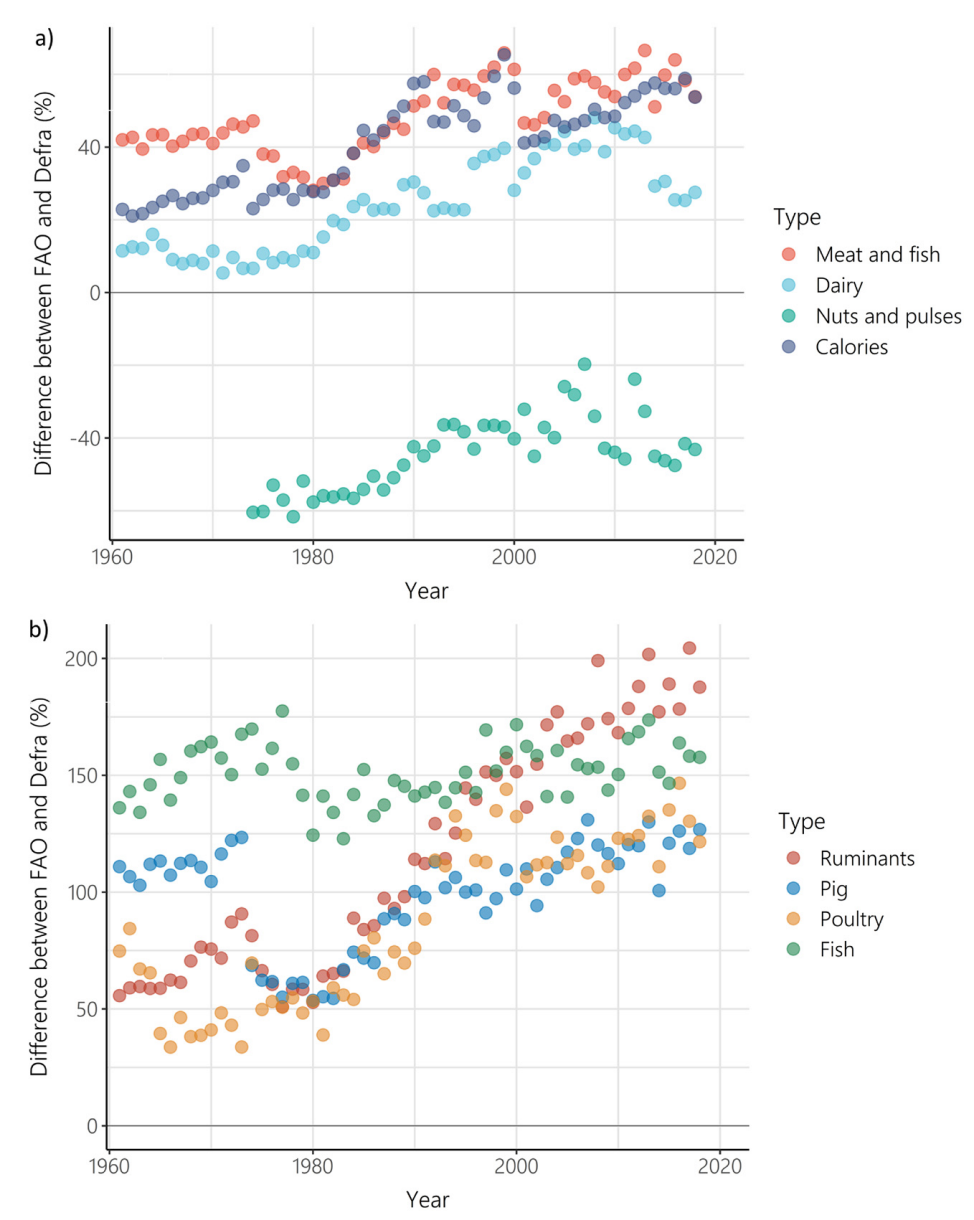
Difference between Food and Agriculture Organisation- food balance sheets (FAO-FBS) food supply and Defra-household budget surveys (Defra-HBS) food purchases between 1961 and 2018 for **a**) meat and fish (p<0.001, r= 0.72, number of years = 58), dairy (p<0.001, ρ = 0.84, number of years = 58), nuts and pulses (p<0.001, r = 0.69, number of years = 45) and calorie provision (p<0.001, ρ = 0.85, number of years = 58), and **b**) ruminants (p<0.001, ρ = 0.93, number of years = 58), pork (p<0.01, ρ = 0.37, number of years = 58), poultry (p<0.001, ρ = 0.82, number of years = 58), and fish (p>0.05, r = 0.22, number of years = 58). Differences greater than 0 indicate that FAO-FBS had higher per capita outputs than Defra-HBS.

While a difference between supply and purchase data is to be expected, the difference between FAO-FBS food supply and Defra-HBS food purchases increased between 1961 and 2018 ( Figure 4). for all food types (other than nuts and pulses), and for calorie intake. For nuts and pulses there was again a positive relationship between the difference between FAO-FBS food supply and Defra-HBS food purchases and time, but as FAO-FBS supply of nuts and pulses was lower than Defra-HBS purchases at the start of the timeseries, FAO-FBS supply and Defra-HBS purchases converged over time. When disaggregated by individual meat types, the difference between FAO-FBS and Defra-HBS increased over time for all meat types (ruminants, pork and poultry), but not for fish, which showed no change over time.

## Discussion

The per capita quantities of food supplied in the UK (as reported by FAO-FBS) and purchased (as reported by Defra-HBS) are significantly different for all food types and for calorie consumption. This difference is greatest for meat and fish, where, on average, FAO-FBS are 48% greater than Defra-HBS. These differences affected the long-term trends in food consumption observed in the UK, as trends in consumption of nuts and pulses and calorie intake did not agree between FAO-FBS and Defra-HBS, and while the trends in meat and fish and dairy consumption did agree, the slope of the trends differed between the two data sources. The difference between FAO-FBS and Defra-HBS was not constant over time, and for all food types and calorie intake (other than nuts and pulses and fish) FAO-FBS and Defra-HBS values diverged between 1961 and 2018.

Our finding that FAO-FBS estimate higher consumption than Defra-HBS is consistent with similar studies within Europe ( Naska *et al*., 2009; Serra-Majem *et al*., 2003) and across the world ( Del Gobbo *et al*., 2015; Grünberger, 2014; Serra-Majem *et al*., 2003). For example Serra-Majem *et al.* (2003) found that FBS overestimate HBS meat consumption by 48% and dairy consumption by 33%. Del Gobbo *et al*. (2015) found that FBS overestimate individual-based Global Dietary Database national dietary intake estimates of meat consumption by 120% and dairy consumption by 173%. While Serra-Majem *et al.* (2003) found that FBS also overestimate HBS nuts and oil seed consumption by 183%, Del Gobbo *et al.* (2015) found that FBS consumption of nuts and seeds were 27% lower than found from dietary surveys, in agreement with our findings. Del Gobbo *et al.* (2015) suggest this could be due to home or local production, or other food sources not captured by FAO-FBS. Rate of underreporting may not be consistent between food types ( Mendez *et al*., 2004) and could be responsible for differences in the extent of disparity observed between FAO-FBS and Defra-HBS. Hirvonen *et al.* (1997) showed that overreporting of ‘healthy’ foods occurs, perhaps partly explaining the overprediction of nuts and pulses by Defra-HBS relative to FAO-FBS.

The differences between reported consumption by FAO-FBS and Defra-HBS could be due to genuine differences in supply and purchases of food, or inaccuracies in data collection. FAO-FBS food supply estimates include retail food waste ( Poore & Nemecek, 2018), whereas Defra-HBS food purchases do not, so some of the observed differences could be due to retail food waste. If so, we would expect greater discrepancies in food with a short shelf-life ( Parfitt *et al*., 2010). However, while meat, fish and dairy usually have a shorter shelf life than nuts and pulses ( Premavalli, 2000), the latter exhibit a greater difference between mean FAO-FBS food supply and mean Defra-HBS food purchases. It seems likely that inaccuracies in data collection, primarily due to under-reporting, are also important. The National Food and Dietary Survey (a HBS similar to Defra’s but run by Public Health England and the UK Food Standards Agency; Public Health England, 2020) has been shown to underestimate calorie consumption. Reported energy intake was shown to be 34% less than energy expenditure (measured using doubly labelled water), giving an indication of substantial under-reporting in the National Food and Dietary Survey ( Office for National Statistics, 2016). A similar level of under-reporting in Defra surveys would account for most of the difference seen between FAO-FBS and Defra-HBS data.

As well as differences in estimates of overall per capita consumption we found no agreement between trends over time in FAO-FBS food supply and Defra-HBS food purchases of calories and nuts and pulses. As such, drawing conclusions about changes in the quantity of these foods consumed is challenging. Quantification of under-reporting and household and retail waste might reveal whether the divergence between FAO-FBS and Defra-HBS-based estimates are due to genuine divergences in supply and purchases or inaccuracies in data collection. Retail waste increased by 6% between 2015 and 2018 ( WRAP, 2020), yet only represented 2.4% of post farm waste in 2015 ( British Retail Consortium, 2016). Data on long-term trends on UK retail waste are limited. Harper & Hallsworth (2015) suggest that under-reporting has increased over time and is responsible for falling calorie intake over time. They propose that increasing obesity levels, increase in desire to lose weight, increased eating outside the home and snacking, falling response rates of surveys and growing disparities between reference data and true portion sizes or food energy density, are responsible for the increase in under-reporting. Here we show that increase in under-reporting may not be limited to calorie intake, as trends in calorie intake differences between FAO-FBS and Defra-HBS are comparable to those of meat and fish, and dairy. These results suggest that the composition of the UK diet should be informed by both FAO-FBS and Defra-HBS data, with knowledge of their limitations.

Stewart *et al*. (2021) found that according to the National Diet and Nutrition Survey (NDNS), meat consumption in the UK declined by 17.4% (103.7g to 86.3g) between 2008/9 and 2018/19. This is a smaller quantity of meat and a larger decline than the trend we observed from Defra-HBS (3.0% decline in meat intake (155.5g to 150.9g) between 2008 and 2018) and FAO-FBS (5.2% decline in meat intake (223.3g to 211.6g) between 2008 and 2018). Both NDNS and Defra-HBS are likely to be subject to under-reporting, unlike FAO-FBS ( Office for National Statistics, 2016). Defra-HBS includes household food waste (as food purchases are measured) and NDNS does not (as food consumption is measured). However, if household food waste were the only discrepancy between the two datasets, this would indicate 42.8% of purchased meat in UK households is wasted (2018: (150.9-86.3)/150.9 = 42.8%), which is substantially higher than the estimated figure for meat and fish household waste of 21% (13.% avoidable (for example, not used in time) and 7.6% unavoidable (for example, bones and fish heads)) ( Quested *et al*., 2013; Quested & Murphy, 2014). This suggests methodology discrepancies are also contributing to this difference.

Defra-HBS estimated steeper falls in ruminant and pork consumption than FAO-FBS and a smaller increase in poultry consumption. It is key to be able to compare consumption of different meat types, given the higher environmental impacts of beef compared to chicken ( Poore & Nemecek, 2018) and the increased disease burden from increased red and processed meat consumption ( Chung *et al*., 2021). While this is straightforward for FAO-FBS, it is hard to accurately sum up for the Defra-HBS data due to categories such as meat pies, ready meals and burgers and “takeaway miscellaneous meats”.

For all food types except nuts and pulses (where the difference over time decreased), the difference between FAO-FBS and Defra-HBS increased between 1961 and 2018. With limited long-term data on the prevalence of under-reporting and extent of waste ( FAO, 2001) identifying reasons for this divergence is challenging. To our knowledge, the increasing difference between FAO-FBS food supply and Defra-HBS food purchases has not been documented before. Due to the problems the divergence between these datasets poses for accurate monitoring of UK dietary change, identifying the reasons for this is an important topic for future research.

Studying the temporal aspect of discrepancies between FAO-FBS and Defra-HBS was a strength of this study. Here we show that the increasing disparity between FBS and HBS in calorie intake found by Harper & Hallsworth (2015) over time is also present for specific food types. A limitation of this study was that we did not quantify the relative contribution of underreporting, retail waste and other differences in data collection methods to the reported discrepancies. This means that the extent to which inconsistencies are attributable to a genuine difference between supply and purchases, or inaccuracies in data collection, processing, and reporting is unknown. Understanding the reasons for discrepancies between FAO-FBS and Defra-HBS reported here, is important for resolving data inaccuracies and improving consistency of dietary monitoring in the UK. Exploration of whether such inconsistencies are also present for macronutrients such as protein and fat may be informative, and yield implications for monitoring progress towards healthier diets.

Efforts to make food systems healthier and more sustainable rely on routinely collected data such as FAO-FBS and Defra-HBS ( Marshall *et al*., 2021). The inconsistencies between these datasets – and the challenges in directly comparing them – raises concerns for evidence-based policy making. The National Food Strategy ( Dimbleby, 2020) recommends creation of a National Food System data programme to monitor and shape progress towards a better food system. While the envisaged collection of data on land use, retail and environmental and health impacts of food outlined in the National Food Strategy will be a vital resource to solve problems in the UK food system, these efforts may be undermined by the inconsistencies between datasets used to monitor UK food supply and purchases, outlined here. High quality surveys are most common in high income countries such as the UK. If these data inconsistencies exist in the UK, they are likely to exist in other countries as well, as exemplified by Del Gobbo *et al*. (2015), Grünberger (2014) and Serra-Majem *et al*. (2003). This raises the question as to whether other methods of data collection are needed for monitoring progress of food systems towards health and sustainability goals, and how inconsistencies in long-term dietary datasets can be reconciled.

## Concluding remarks

Data produced on food supply by FAO-FBS and on food purchases by Defra-HBS differ for all food types and for calories, both overall and in terms of temporal trends. The difference between FAO-FBS and Defra-HBS increased over the time period studied, raising questions about the reliability of both data sources for monitoring dietary change, especially when used as routine data sources for evidence-based decision-making. Further research concerning the reasons for disagreement between FAO-FBS and Defra-HBS is required.

## Data availability

### Underlying data

University of Cambridge Repository: Research data supporting "Discrepancies between two long-term dietary datasets in the United Kingdom (UK)". https://doi.org/10.17863/CAM.78207 ( Smith *et al*., 2021).

- DataFrom_Figures_2_3_4_Tables_2_3.xlsx
- Household consumption of selected foods from 1942 to 2000 – 1942-2000.csv
- Household nutrient data from 1940 to 2000 – 1940-2000.csv
- UK - eating out purchases.ods
- UK - household and eating out nutrient intakes.ods
- UK - household purchases.ods

Data are available under the terms of the Creative Commons Attribution 4.0 International license (CC-BY 4.0).

## Source data

### FAO-FBS data

Data for between 1961 and 2018 are available from FAOSTAT and were first downloaded on 08/01/2020 from http://www.fao.org/faostat/en/#data/FBSH titled *'Food Balances (-2013, old methodology and population)*'.

- *Data on food supply* was downloaded by selecting *'United Kingdom of Great Britain and Northern Ireand'* from *'Countries' and 'Food supply quantity (kg/capita/yr)'* from *'Elements'* between 1961 and 2013 for the items listed in Table 1.
- *Data on calorie supply* was downloaded by selecting *'United Kingdom of Great Britain and Northern Ireand'* from *'Countries'*, *'Food supply quantity (kcal/capita/day)*' from *'Elements'*, and *'Grand Total + (Total)* from *'Items (aggregated)'* between 1961 and 2013.

Data for between 2014 and 2018 are available from FAOSTAT and were first downloaded on 24/10/2021 from https://www.fao.org/faostat/en/#data/FBS titled *'Food Balances (2014-)'.*

- *Data on food supply* was downloaded by selecting *'United Kingdom of Great Britain and Northern Ireand*' from ' *Countries*' and *'Food supply quantity (kg/capita/yr)*' from ' *Elements*' between 2014 and 2018 for the items listed in Table 1.
- *Data on calorie supply* was downloaded by selecting *'United Kingdom of Great Britain and Northern Ireand*' from ' *Countries*', *'Food supply quantity (kcal/capita/day)*' from ' *Elements*', and *'Grand Total + (Total)'* from *'Items (aggregated)'* between 2014 and 2018.

Data are available under the terms of the Creative Commons Attribution-NonCommercial-ShareAlike 3.0 IGO (CC BY-NC- SA 3.0 IGO).

### Defra-HBS data

Family Food Module data and adjusted National Food Survey data were first downloaded on 19/02/2020 from https://www.gov.uk/government/statistical-data-sets/family-food-datasets.

- Datasets are named ' *UK - household purchases*', ' *UK - eating out purchases*' and ' *UK - household and eating out nutrient intakes*'.

National Food Survey data were downloaded on 08/01/2020 from https://webarchive.nationalarchives.gov.uk/20130103024837/http://www.defra.gov.uk/statistics/foodfarm/food/familyfood/nationalfoodsurvey/.

- National Food Survey data are named ' *Household nutrient data from 1940 to 2000 – 1940-2000*' and ' *Household consumption of selected foods from 1942 to 2000 – 1942-2000*'.

Data are available under the terms of the Open Government Licence v3.0.
