# Supplementary material for: Discrepancies between two long-term dietary datasets in the United Kingdom (UK)
Source: Wellcome Open Res. 2023 Jan 11;6:350. Originally published 2021 Dec 16. [Version 3] doi: 10.12688/wellcomeopenres.17245.3 (PMC9494176; doi:10.12688/wellcomeopenres.17245.3)
Supplement: Peer review history

# Peer review: Reviewer response for version 1

**DOI:** 10.21956/wellcomeopenres.19061.r50555

**Published:** 2022-06-24

**Authors:** Paul West

**Affiliations:** 1 Institute on the Environment, University of Minnesota, Minneapolis, MN, USA; 2 Project Drawdown, San Francisco, CA, USA

Smith and colleagues provide a solid analysis that compares the two main databases--FAO's Food Balance Sheets and DEFRA's Household Budget Surveys--for assessing dietary status and trends. The discrepancies, how the change over time, and potential sources of these discrepancies are well explained. Their results have important implications for assessing trends and setting science-based policies related to diet in the UK. 

 The paper is sufficient for indexing as it is at the time of my review (24 June 2022), but I think it can be improved by discussing the implications and recommendations. Here are a few examples of topics that would strengthen the discussion:

1. Is one of the databases better suited for some analyses than others? Is the FAO database better for assessing the environmental impacts of diet as it includes food waste? Is the HBS better suited for assessing connections between household-level choices and their relation to demographics and price changes? 
2. Do you recommend that most analysis used for research or policy use both databases? If so, is one better in terms of totals (FAO if HBS under-reports??) and then use the relative trends in both databases to present a range? For example, it looks like FAO estimates of g/capita/day of meat consumption are 2-3x estimates from HBS (Fig 3).
3. You mention that food waste explaining some of the difference in the values because it is embedded in the FAO data. But if 70% of the post farmgate waste in the UK is at the consumer stage, waste doesn't explain the much of differences in the databases, right?
4. What are ways to improve the data? What recommendations of either future changes in data collection or post processing (but still allow for consistency across the time series)? Could you get closer to the "truth" by adding a third data set, such as national-level sales data for foods like beef? 
5. In a few places in the paper, other studies are cited that noted that the differences in the databases "can be highly problematic" (Del Gobbo *et al.* 2015, Sarra-Majem *et al. *2003). Did these authors address any of the above ideas related to implications or recommendations? If so, how does your analysis and assessment of implications compare? In other words, provide more detail than "it's problematic."

Minor point:

- The Excel Version is noted as 2108, which I assume is meant to be 2018.

Is the work clearly and accurately presented and does it cite the current literature?

Yes

If applicable, is the statistical analysis and its interpretation appropriate?

Yes

Are all the source data underlying the results available to ensure full reproducibility?

Yes

Is the study design appropriate and is the work technically sound?

Yes

Are the conclusions drawn adequately supported by the results?

Yes

Are sufficient details of methods and analysis provided to allow replication by others?

Yes

Reviewer Expertise:

food systems, ecosystems, global change

I confirm that I have read this submission and believe that I have an appropriate level of expertise to confirm that it is of an acceptable scientific standard.

# Response

**Published:** 2022-08-28

**Authors:** Kerry Smith

**Affiliations:** University of Reading, UK

**Comment 1.**

 Smith and colleagues provide a solid analysis that compares the two main databases--FAO's Food Balance Sheets and DEFRA's Household Budget Surveys--for assessing dietary status and trends. The discrepancies, how the change over time, and potential sources of these discrepancies are well explained. Their results have important implications for assessing trends and setting science-based policies related to diet in the UK.

 The paper is sufficient for indexing as it is at the time of my review (24 June 2022), but I think it can be improved by discussing the implications and recommendations.

**Response 1.**

 We are grateful to the reviewer for these positive comments, and for the suggestions on how to improve the discussion. We address the suggestions individually below.

**Comment 2.**

 Here are a few examples of topics that would strengthen the discussion: Is one of the databases better suited for some analyses than others? Is the FAO database better for assessing the environmental impacts of diet as it includes food waste? Is the HBS better suited for assessing connections between household-level choices and their relation to demographics and price changes?

 Do you recommend that most analysis used for research or policy use both databases? If so, is one better in terms of totals (FAO if HBS under-reports??) and then use the relative trends in both databases to present a range? For example, it looks like FAO estimates of g/capita/day of meat consumption are 2-3x estimates from HBS (Fig 3).

**Response 2.**

 We thank the reviewer for the suggestions. We added the following paragraph to the discussion,

*“With current data quality and availability, we recommend that where possible, both FAO-FBS and Defra-HBS are used in parallel to monitor dietary trends. For specific applications, use of one dataset may be appropriate. For example, when conducting assessments of the environmental impact of food consumption, the presence of underreporting in Defra-HBS risks underestimation of the environmental impact of UK food consumption. In contrast, food balance sheets (FAO-FBS) are not subject to underreporting, and are readily conciliable with major life cycle analyses datasets (Poore and Nemecek, 2018), so are best suited to this application. The inclusion of retail waste in FAO-FBS estimates impedes monitoring of purchasing patterns. Household budget surveys (Defra-HBS) monitor purchases directly, and are published alongside demographic data, so are appropriate for monitoring changes in purchasing patterns, and comparisons between demographic groups. Understanding dataset methodology and limitations supports decision making in UK dietary analyses, when identifying the most suitable dataset or datasets to use.”*

**Comment 3.**

 You mention that food waste explaining some of the difference in the values because it is embedded in the FAO data. But if 70% of the post farmgate waste in the UK is at the consumer stage, waste doesn't explain the much of differences in the databases, right?

**Response 3.**

 In agreement with the reviewer, we believe that waste doesn’t explain much of the differences in the datasets, due to its small contribution to total post farm waste. As such we have edited the discussion accordingly,

*“Retail waste increased by 6% between 2015 and 2018* *(WRAP, 2020)* *, yet only represented 2.4% of post farm waste in 2015* *(British Retail Consortium, 2016)* *. Data on long-term trends on UK retail waste are limited, but given its small contribution to total post farm waste, it’s unlikely waste is a major factor causing the divergence between FAO-FBS and Defra-HBS.”*

**Comment 4.**

 What are ways to improve the data? What recommendations of either future changes in data collection or post processing (but still allow for consistency across the time series)? Could you get closer to the "truth" by adding a third data set, such as national-level sales data for foods like beef?

**Response 4.**

 We have added the following to the discussion.

 “ *Analyses of UK dietary trends are impeded by the differences between FAO-FBS and Defra-HBS. One approach, used by Del Gobbo et al. (2015), is to apply calibration models to adjust FAO-FBS to dietary surveys. This assumes that dietary surveys provide an accurate estimation of consumption, which given the observed levels of underreporting in the National Food and Dietary Survey* *(Office for National Statistics, 2016)* *, may not be the case. To improve understanding of UK dietary trends, monitoring of underreporting across time and food types is necessary. Additionally, data on the proportion of food wasted at each stage of the production chain, disaggregated by food type, would assist in reconciling differences between FAO-FBS and Defra-HBS. If data collection on waste and underreporting were integrated into current data collection processes, and published alongside dietary datasets, this would allow uncertainties to be reduced, whilst retaining consistency in long term temporal monitoring. Improving data availability on waste and underreporting is key for enabling robust analyses of UK dietary change.”*

**Comment 5.**

 In a few places in the paper, other studies are cited that noted that the differences in the databases "can be highly problematic" (Del Gobbo et al. 2015, Sarra-Majem et al. 2003). Did these authors address any of the above ideas related to implications or recommendations? If so, how does your analysis and assessment of implications compare? In other words, provide more detail than "it's problematic."

**Response 5.**

 We expanded on the implications and recommendations made by other studies comparing food balance sheet and household budget surveys, in the introduction,

 “ *Findings from studies outside the UK indicate that using only one of these methodologies to assess dietary trends can be highly problematic (Del Gobbo et al., 2015;* Serra-Majem et al., 2003) , *and hinders food policy planning*, which *requires accurate knowledge of food consumption patterns (* *Serra-Majem et al., 2003)* *.* *Investigating the* *investigating reasons behind data discrepancies helps to understand their improves understanding of the limitations of these datasets* *(Benthem de Grave et al., 2020)* *.”*

 We also added detail to the discussion.

*“ Analyses of UK dietary trends are impeded by the differences between FAO-FBS and Defra-HBS. One approach, used by Del Gobbo et al. (2015), is to apply calibration models to adjust FAO-FBS to dietary surveys. This assumes that dietary surveys provide an accurate estimation of consumption, which given the observed levels of underreporting in the National Food and Dietary Survey* *(Office for National Statistics, 2016)* *, may not be the case. To improve understanding of UK dietary trends, monitoring of underreporting across time and food types is necessary.”*

**Comment 6.**

 The Excel Version is noted as 2108, which I assume is meant to be 2018.

**Response 6.**

 We have doubled checked the Excel Version number and it is supposed to be 2108.

# Peer review: Reviewer response for version 1

**DOI:** 10.21956/wellcomeopenres.19061.r47702

**Published:** 2022-01-17

**Authors:** Dora Marinova

**Affiliations:** 1 Curtin University Sustainability Policy (CUSP) Institute, Curtin University, Perth, WA, Australia

This is a very important and timely analysis. It also has been carried out in a competent manner. The conclusions that the authors draw are equally important. 

 Being the first paper that specifically tackles the differences between different databases and surveys, it also demonstrates the challenges that food supply and consumption face in terms of data collection and coverage.

 I have no hesitation to recommend for this paper to be published. Below are some recommendations which I think will approve the readability of the paper for an international audience:

- DEFRA stands for Department for Environment and Rural Affairs (which is not stated) and the acronym should be fully capitalised.
- There are a couple of 2008-2018 decreasing trends for meat and fish in both databases. It would be good if they are also presented graphically.
- The discussion section would benefit from some possible explanations for the discrepancies in nuts and legumes in the case of UK.
- The conclusion section could be expanded to do justice to the study. For example, it is important to emphasise again the issue of waste. Highlights from the findings can also be included.
- Some minor editorial comments – in academic writing sentences should not start with an acronym; the verb in relation to FAO should be used in singular referring to the organisation.

Is the work clearly and accurately presented and does it cite the current literature?

Yes

If applicable, is the statistical analysis and its interpretation appropriate?

Yes

Are all the source data underlying the results available to ensure full reproducibility?

Yes

Is the study design appropriate and is the work technically sound?

Yes

Are the conclusions drawn adequately supported by the results?

Yes

Are sufficient details of methods and analysis provided to allow replication by others?

Yes

Reviewer Expertise:

sustainability, food systems

I confirm that I have read this submission and believe that I have an appropriate level of expertise to confirm that it is of an acceptable scientific standard.

# Response

**Published:** 2022-08-28

**Authors:** Kerry Smith

**Affiliations:** University of Reading, UK

**Comment 1:**

 This is a very important and timely analysis. It also has been carried out in a competent manner. The conclusions that the authors draw are equally important.

 Being the first paper that specifically tackles the differences between different databases and surveys, it also demonstrates the challenges that food supply and consumption face in terms of data collection and coverage.

 I have no hesitation to recommend for this paper to be published. Below are some recommendations which I think will approve the readability of the paper for an international audience.

**Response 1:**

 We are grateful to the reviewer for their positive comments and recommendations. We address them individually below.

**Comment 2:**

 DEFRA stands for Department for Environment and Rural Affairs (which is not stated) and the acronym should be fully capitalised.

**Response 2:**

 We have added the full name for Defra in the abstract and in first use in the introduction.

 In the abstract,

*“We used UK longitudinal dietary data on food supply, provided by the Food and Agriculture Organisation (FAO) (FAO-FBS, 1961-2018), and food purchases, provided by the Department for Environment, Food and Rural Affairs (Defra) Defra (Defra-HBS, 1942-2018).”*

 In the introduction,

 “ *Both food balance sheets (FBS) (published by Food and Agriculture Organisation (FAO), hereafter referred to as FAO-FBS) and household budget surveys (HBS) (published by the Department for Environment, Food and Rural Affairs (Defra) Defra in the United Kingdom (UK), hereafter referred to as Defra-HBS) have been used to approximate trends in consumption over time (for example* *Grünberger, 2014* *and* *Peng et al., 2015)* *.*”

 The UK government does not capitalise Defra so we have followed the same formatting.

**Comment 3:**

 There are a couple of 2008-2018 decreasing trends for meat and fish in both databases. It would be good if they are also presented graphically.

**Response 3:**

 We have added trend lines between 2008-2018 (where significant) to figure 2, and added a paragraph to explain these trends in the results.

*“* *Long-term trends in consumption were not always representative of short-term trends (Figure 3, Tables 2 and 4). Between 2008 and 2018, both FAO-FBS and Defra-HBS estimated significant declines in meat consumption (FAO-FBS -5.2%, Defra-HBS -3.0%), in contrast to the significant increase estimated by both datasets over the whole time period (Table 3). Significant declines in dairy supply between 2008-2018 (-15.0%) were estimated by FAO-FBS despite no significant trend between 1961 and 2018. No significant change in supply of nuts and pulses and calorie intake was estimated by FAO-FBS between 2008 and 2018, despite significant increases in supply between 1961 and 2018.* *”*

**Comment 4:**

 The discussion section would benefit from some possible explanations for the discrepancies in nuts and legumes in the case of UK.

**Response 4:**

 While we have provided some explanation for why the difference in estimations between FAO-FBS and Defra-HBS for nuts and pulses might differ from other food groups, we did not provide information on why FAO-FBS and Defra-HBS might be converging for nuts and pulses and not other food groups. As such, we have added the following to the discussion.

*“ Nuts and pulses are an exception to the observed divergence, with FAO-FBS supply and Defra-HBS purchases converging overtime. This could be due to an increase in home production of food since 1985 (Defra, 2021), or a decrease in under-reporting (for example due to the rise in awareness of the environmental benefit of switching to plant-based protein (Alae-Carew et al., 2022)).”*

**Comment 5:**

 The conclusion section could be expanded to do justice to the study. For example, it is important to emphasise again the issue of waste. Highlights from the findings can also be included.

**Response 5:**

 We have expanded the conclusions section as below.

*“Data produced on food supply by FAO-FBS and on food purchases by Defra-HBS differ for all food types and for calories, both overall and in terms of temporal trends. Underreporting and retail waste were the main reasons for these differences, with underreporting expected to be the greatest contributor. Further research concerning the reasons for disagreement between FAO-FBS and Defra-HBS is required. Specifically, data collection on temporal trends in underreporting, and food waste at each stage of the production chain, disaggregated by food type, could assist in reconciling the differences between datasets.*

*We recommend that where possible assessments of dietary trends use both household budget surveys and food balance sheets in parallel, with knowledge of their limitations. For most food groups, the difference between FAO-FBS and Defra-HBS increased over time raising questions about the reliability of both data sources for monitoring dietary change, especially when used as routine data sources for evidence-based decision-making.”*

**Comment 6:**

 Some minor editorial comments – in academic writing sentences should not start with an acronym; the verb in relation to FAO should be used in singular referring to the organisation.

**Response 6:**

 Sentences starting with an acronym have been rearranged.
